# Exploring the rumen fluid metabolome using liquid chromatography-high-resolution mass spectrometry and Molecular Networking

**DOI:** 10.1038/s41598-018-36196-4

**Published:** 2018-12-19

**Authors:** Rafaela Takako Ribeiro de Almeida, Rodolpho Martin do Prado, Carla Porto, Geraldo Tadeu dos Santos, Sharon Ann Huws, Eduardo Jorge Pilau

**Affiliations:** 1 LaBioMass, Chemistry Department, Universidade Estadual de Maringá, Maringá, Brazil; 2 Animal Science Department, Universidade Estadual de Maringá, Maringá, Brazil; 3 Program of Master in Science, Technology and Food Safety and Cesumar Institute of Science, Technology and Innovation - ICETI. University Center of Maringá - UNICESUMAR, Maringá, Brazil; 4 Institute for Global Food Security, Queen’s University of Belfast, Northern Ireland, UK

## Abstract

The rumen primary and secondary metabolite content is intimately related to its community of bacteria, protozoa, fungi, archaea and bacteriophages, ingested feed and the host. Despite the myriad of interactions and novel compounds to be discovered, few studies have explored the rumen metabolome. Here, we present the first study using ultra-high performance liquid chromatography tandem mass-spectrometry and Molecular Networking approach, and various extraction methods on the cell-free rumen fluid of a non-lactating Holstein cow. Putative molecules were annotated based on accurate fragmentation matching the Global Natural Products Social Molecular Networking library, public spectral libraries, or annotated manually. The combination of five extraction methods resulted on 1,882 molecular features observed. Liquid-liquid extraction resulted on the highest molecular features abundance, 1,166 (61.96% of total). Sixty-seven compounds were annotated using Global Natural Products Social Molecular Networking library and public libraries, such as hydrocinnamic and azelaic acid, and monensin. Only 3.56% of molecular features (67) observed had positive match with available libraries, which shows the potential of the rumen as reservoir of novel compounds. The use of untargeted metabolomics in this study provided a snapshot of the rumen fluid metabolome. The complexity of the rumen will remain long unknown, but the use of new tools should be encouraged to foster advances on the rumen metabolome.

## Introduction

The rumen is a complex anaerobic environment enriched in bacteria, protozoa, fungi, archaea and bacteriophages^1^. The symbiotic relationship between the host and the microbiota results on the transformation of human non-edible feedstuff into high-quality products, such as meat and milk. The degradation of feed material in the rumen is a dynamic and continuous process orchestrated by a myriad of microbial enzymes, and this process is affected by the input and output of feed and water, ruminal pH and motility, and others^2^. Exploring the rumen microbial processes, interactions and functionality is key to improve feed efficiency, decrease methane emission, and to discover novel enzymes and antimicrobials^3–5^.

Recent studies using next-generation sequencing have provided evidence on the abundance of microorganisms and genes present in the rumen^3,6^. However, there is little information on the chemical composition of secondary microbial metabolites and the expressed proteins in the rumen. The first study to employ different analytical methods, including mass spectrometry (MS), to explore the rumen metabolome was performed by Saleem *et al*.^7^ The rumen fluid of 8 dairy cows fed different amounts of barley were evaluated and a total of 93 molecular features were observed. To further characterize the rumen fluid metabolome, Saleem *et al*.^8^ identified and quantified routinely detected metabolites such as amino acids, carbohydrates, organic acids and others. A total of 246 molecular features were identified, of which 116 were derivatized and extracted using a commercial kit and analyzed using direct flow injection tandem MS. Recently, Artegoitia *et al*.^9^ analyzed rumen fluid collected after slaughter of beef cattle using liquid chromatography-mass spectrometry (LC-MS) to explore potential metabolite markers related to average daily gain. Thirty-three metabolites were reported to be associated with differences on average daily gain.

Metabolome investigations require a comprehensive approach to represent the molecular features present in a sample. The association between extraction methods, analytical techniques and annotation tools are essential to cover metabolites from various chemical classes, especially for complex samples. The recent developed annotation tool Molecular Networking (MN) based in MS allows users to visually and structurally evaluate related metabolites with similar fragmentation patterns^10,11^. Thus, molecular families can be clustered within groups on a chemical map, providing the possibility to comprehensively interpret large metabolomics datasets.

In this study, Liquid-Liquid Extraction (LLE), Solid Phase Extraction (SPE) and 3 variations of the Quick, Easy, Cheap, Effective, Rugged, and Safe (QuEChERS) extraction method were used to provide a snapshot of the rumen fluid metabolome of a non-lactating dairy cow. Different extraction methods aimed to extract metabolites varying in chemical properties, such as polarity and p*K*a. Also, we present the first study using MS-based MN approach to explore the rumen fluid metabolome.

## Material and Methods

### Chemicals

Sodium acetate (NaOAc.3H_2_O), magnesium sulphate (MgSO_4_, anhydrous), sodium sulphate (Na_2_SO_4_, anhydrous), sodium chloride (NaCl) and acetic acid were obtained from Synth (Diadema, SP, Brazil). Primary-secondary amine (PSA) and silica-based sorbent with C18 bonding (C18) were obtained from Supelco (Bellefonte, PA, USA). Solid-phase extraction cartridges were obtained from Applied Separations (Allentown, PA, USA) and filled using C18 phase (Supelco; Bellefonte, PA, USA) and Cyano phase (CN; Phenomenex; Torrance, CA, USA). Ethyl acetate was obtained from Dinâmica (Diadema, SP, Brazil). Water with 0.1% formic acid (v:v; MS grade), formic acid (≥96% purity) and ammonium hydroxide solution (28–30%; NH_3_ basis) were obtained from Sigma-Aldrich (St. Louis, MO, USA). Methanol and acetonitrile of UHPLC grade were purchased from J.T. Baker (Phillipsburg, NJ, USA). Ultra-pure water was produced using an EMD Millipore Direct-Q™ 3 system (Merck Millipore; Burlington, MA, USA). Mixed cellulose esters membrane filter (47 mm diameter with 0.22 and 0.45 µm pore size) and glass vacuum filter holder for 47 mm disc filters were obtained from Merck Millipore (Burlington, MA, USA).

### Animal and diet

A multiparous non-lactating Holstein cow with 572 kg of body weight fitted with a ruminal cannula (10 cm; Bar Diamond Inc., Parma, ID, USA) was used. All animal care and experimental procedures were conducted under the surveillance of the Animal Care and Use Committee of the Universidade Estadual de Maringá, Brazil (protocol no. 9013160518) and met the guidelines of the National Council for the Control of Animal Experimentation (CONCEA). Diet was offered for 21 days and consisted, on a DM basis, of 60% corn silage, 24% of corn grain, 10% of wheat bran, 4.8% of soybean meal, and 1.2% of mineral mixture (which contained 480 mg/kg of monensin). The cow was fed at 07 h 00 and 15 h 00 for *ad libitum* intake and was housed in individual stall with free access to clean water.

### Rumen fluid sampling

At day 21, rumen content was sampled (300 mL) through a ruminal cannula 10 min before the morning feeding. The rumen content was filtered through 4 layers of cheesecloth into a glass amber container on ice. The pH of the rumen fluid was determined immediately after sampling using a pH meter (6.6; Tecnal, SP, Brazil). Filtered rumen fluid was centrifuged within 30 minutes at 1,000 *g* for 5 min at 4 °C, the pellet was discarded, and the supernatant was subsequently centrifuged at 13,000 *g* for 30 min at 4 °C. The cell-free supernatant was then filtered using 0.45 µm pore size membranes, and subsequently using 0.22 µm pore size membranes, both using a Millipore filtering system. Aliquots were stored at −20 °C for one day until extraction procedures were performed.

### Liquid-liquid extraction of rumen fluid metabolites

One aliquot of cell-free rumen fluid (10 mL) was mixed with 10 mL of ethyl acetate and 1.0 g of NaCl for the LLE (Supplementary Fig. S1). The solution was vortexed for 2 min and allowed to separate into two phases. The upper organic layer was decanted, and the bottom layer was mixed with another 10 mL of ethyl acetate and 1.0 g of NaCl. This procedure was repeated one more time. The organic phases were combined and concentrated under nitrogen flow (LLE-1). Similar procedures were repeated using ethyl acetate and 1% acetic acid (v:v; LLE-2), ethyl acetate and 1% ammonium hydroxide (v:v; LLE-3) instead of ethyl acetate. Extracts were analyzed using ultra-high performance liquid chromatography–tandem mass-spectrometry (UHPLC-MS/MS).

### Solid-phase extraction of rumen fluid metabolites

Solid-phase extractions (Supplementary Fig. S2) were performed using different solid phase characteristics (C18 and CN) and elution solvents (acetonitrile and methanol). C18 and CN cartridges contained 1 g of sorbent and had 6 mL of reservoir volume. Elution rate was 6 mL min^−1^. C18 cartridges were activated with 5 mL of acetonitrile followed by conditioning with 10 mL H_2_O. Homogenized cell-free rumen fluid (6 mL) was loaded into C18 cartridges and collected for analysis (SPE-1B). The cartridge was washed with 10 mL H_2_O, followed by elution with 6 mL acetonitrile (SPE-1A). C18 cartridges were activated with 5 mL of methanol followed by conditioning with 10 mL H_2_O. Homogenized cell-free rumen fluid (6 mL) was loaded into C18 cartridges and collected for analysis (SPE-2B). The cartridge was washed with 10 mL H_2_O, followed by elution with 6 mL methanol (SPE-2A). CN cartridges were activated with 5 mL of acetonitrile followed by conditioning with 10 mL H_2_O. Homogenized cell-free rumen fluid (6 mL) was loaded into CN cartridges and collected for analysis (SPE-3B). The cartridge was washed with 10 mL H_2_O, followed by elution with 6 mL acetonitrile (SPE-3A). CN cartridges were activated with 5 mL of methanol followed by conditioning with 10 mL H_2_O. Homogenized cell-free rumen fluid (6 mL) was loaded into CN cartridges and collected for analysis (SPE-4B). The cartridge was washed with 10 mL H_2_O, followed by elution with 6 mL methanol (SPE-4A). The 8 SPE aliquots were concentrated under nitrogen flow before UHPLC-MS/MS analysis.

### Original QuEChERS extraction of rumen fluid metabolites

Homogenized cell-free rumen fluid (10 mL) was mixed with an equivalent volume of acetonitrile and vortexed for 1 min for the QuEChERS procedure^12^ (Supplementary Fig. S3). Four g anhydrous MgSO_4_ and 1 g NaCl were added into the solution, which was vortexed for 1 min. The solution was centrifuged for 5 min at 5,000 *g*. The supernatant was collected, 2 mL were split in 2 aliquots for cleanup, and the remainder (5 mL) was concentrated under nitrogen flow (OQ-1). An aliquot of 1 mL of the upper acetonitrile layer was mixed with 150 mg anhydrous MgSO_4_ and 25 mg PSA sorbent, vortexed for 30 s and centrifuged for 1 min at 6,000 *g* (OQ-2). The OQ-2 procedure was repeated, however 25 mg of C18 was used instead of PSA (OQ-3). Extracts were analyzed using UHPLC-MS/MS.

### Buffered QuEChERS extraction of rumen fluid metabolites

Homogenized cell-free rumen fluid (10 mL) was mixed with an equivalent volume of 1% acetic acid in acetonitrile (v:v), 4 g anhydrous MgSO_4_ and 1.7 g NaOAc.3H_2_O, for the buffered QuEChERS procedure^13^ (Supplementary Fig. S4). The solution was vortexed for 1 min and centrifuged for 3 min at 11,000 *g*. The supernatant was collected, 2 mL were split in 2 aliquots for the cleanup step, and the remainder (5 mL) was concentrated under nitrogen flow (BQ-1). A 1 mL aliquot was mixed with 150 mg anhydrous MgSO_4_ and 50 mg sorbent PSA, vortexed for 20 s and centrifuged for 1 min at 6,000 *g* (BQ-2). The BQ-2 procedure was repeated, however 50 mg of C18 was used instead of PSA (BQ-3). Homogenized cell-free rumen fluid (10 mL) was also mixed with an equivalent volume of 1% acetic acid in methanol (v:v), 4 g anhydrous MgSO_4_ and 1.7 g NaOAc.3H_2_O. The solution was vortexed for 1 min and centrifuged for 3 min at 11,000 *g*. The supernatant was split in 2 aliquots of 1 mL for the cleanup step. A 1 mL aliquot was mixed with 150 mg anhydrous MgSO_4_ and 50 mg sorbent PSA, vortexed for 20 s and centrifuged for 1 min at 6,000 *g* (BQ-4). The BQ-4 procedure was repeated, however 50 mg of C18 was used instead of PSA (BQ-5). Extracts were analyzed using UHPLC-MS/MS.

### Acid-base QuEChERS extraction of rumen fluid metabolites

Homogenized cell-free rumen fluid was mixed with 1% acetic acid in acetonitrile (v:v) and 6 g Na_2_SO_4_ anhydrous according to Wang *et al*.^14^ with minor modifications (Supplementary Fig. S5). The solution was vortexed for 2 min, centrifuged for 10 min at 6,000 *g* and the upper layer was collected (acid phase). The bottom layer was mixed with 10 mL solution of 1% ammonium hydroxide in acetonitrile (v:v). The extract was vortexed for 2 min, centrifuged for 10 min at 6000 *g* and the upper layer was collected (basic phase). An aliquot of 5 mL from the acid and basic phases were mixed and the remainder aliquots were concentrated separately under nitrogen flow (ABQ-1 and ABQ-2; respectively). The combined 10 mL aliquot was split in two aliquots. One aliquot was mixed with 0.5 g NaAc, 50 mg C18 and 75 mg PSA sorbent, vortexed for 2 min and centrifuged for 10 min at 6,000 *g* (ABQ-3). The other aliquot was mixed with 0.5 g NaAc and 50 mg C18, vortexed for 2 min and centrifuged for 10 min at 6,000 *g* (ABQ-4). Extracts were analyzed using UHPLC-MS/MS.

### Untargeted metabolomics analysis

Extracts were analyzed using an ultra-high performance liquid chromatograph (Shimadzu, Nexera X2, Japan) coupled to a hybrid quadrupole time-of-flight high resolution mass spectrometer (Impact II, Bruker Daltonics Corporation, Germany) equipped with an electrospray ionization source. Chromatographic separation was performed with an Acquity UHPLC^®^ CSH^TM^ C18 packed with 135 Å pore, 1.7 µm particle size, 2.1 × 100 mm column (Waters, UK), at a flow rate of 0.2 mL min^−1^. The gradient mixture of solvents A (H_2_O with 0.1% formic acid; v:v) and B (acetonitrile with 0.1% formic acid; v-v) was as follow: 5% B 0–1 min, 50% B 1–5 min, 95% B 5–10 min, maintained at 95% B 10–16 min, 5% B 16–18 min, and maintained at 5% B 18–21 min at 40 °C. The capillary voltage was operated in positive and negative ionization modes, set at 4500 and 3000 V, respectively; with an end plate offset potential of −500 V. The dry gas parameters were set to 8 L min^−1^ at 180 °C with a nebulization gas pressure of 4 bar. Data were collected from *m/z* 50 to 1300 with an acquisition rate of 5 Hz, and the 4 ions of interest were selected by auto MS/MS scan fragmentation.

**Figure 1:**
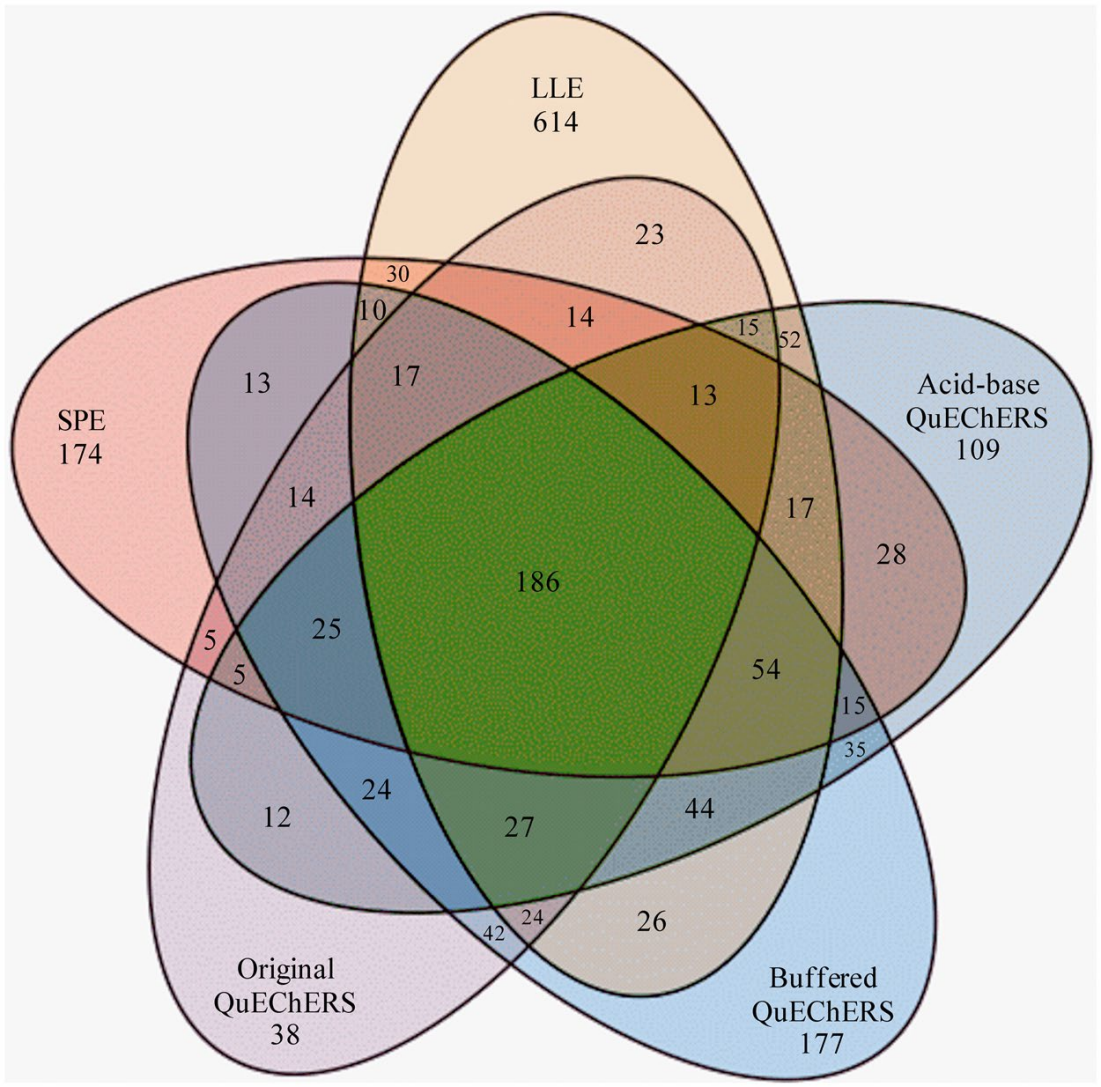
Venn diagram compiling molecular features extracted from the rumen fluid of a non-lactating dairy cow using liquid-liquid extraction; solid-phase extraction; original, buffered and acid-base QuEChERS. Extracts were analyzed using ultra-high performance liquid chromatography coupled to a high resolution mass spectrometer in tandem.

Molecular networking approach required the conversion of mass spectrometry raw data into mzXML file format followed by upload to the Global Natural Products Social Molecular Networking (GNPS) to generate the MN, according to GNPS guidelines^10^ (Supplementary Method S1). The GNPS approach consists of comparing fragmentation spectra (MS/MS experiments) and grouping molecules with similar chemical structures. Each spectrum is represented as a node in the visual MN, and spectrum-to-spectrum alignments are represented as lines that connect nodes, evidencing ions that are correlated with each other. Communication between the nodes are related to similarities in fragmentation spectra between the ions, and similarity between fragmentations patterns are evaluated via vector relation. Structurally related molecules exhibit similar fragmentation patterns; therefore, molecular families tend to unite within groups in the MN, referred as clusters. In addition, it is possible to determine the difference of *m/z* between nodes, defining spectral proximity between all MS/MS spectra in a dataset. This tool innovates and facilitates the analysis of large datasets. It also permits the comparison of molecular features to the GNPS spectral library and all publicly available data. Molecular networking was visualized using Cytoscape^15^. Metabolites with positive match with the GNPS library had both parent and fragment ions manually compared with GNPS spectral library and publicly available data (Supplementary Figs S6–S72). Mass error was lower than 10 ppm (Supplementary Table S1). A Venn diagram was constructed in Microsoft Excel from exported molecular features data using GNPS (Fig. 1).

## Results

### Extraction methods and UHPLC-MS/MS to explore the rumen fluid metabolome

The combination of five extraction methods in the rumen fluid of a non-lactating Holstein cow resulted on 1,882 molecular features analyzed by UHPLC-MS/MS. A total of 67 compounds were identified using publicly available molecular matches (Supplementary Tables S1 and S2), which represents 3.56% of the observed molecular features. Data compiled by extraction method (LLE, SPE, original QuEChERS, buffered QuEChERS and acid-base QuEChERS) was used to generate a Venn diagram (Fig. 1). A total of 186 molecular features (9.88% of total) were present on the five tested extraction methods, of which 27 were identified.

Liquid-liquid extraction had the greatest molecular features abundance: 1,166, which was 61.96% of total. Moreover, LLE extracts had 614 exclusive molecular features (32.62% of total). The LLE also provided the greatest amount of annotated molecular features: 57 of 67. Compounds such histamine and tyramine (Supplementary Table S1), were only present when ammonium hydroxide in ethyl acetate were used (LLE-3). Extractions of syringic acid, decanedioic acid, dodecanedioic acid and 1,11-undecanedioic acid were present when acidified ethyl acetate (LLE-2) was used. Phenacylamine, niranthin and monensin were present on all LLE extracts. The main mammalian enterolignan produced in the rumen, enterolactone (EL), was extracted by the LLE-1, LLE-3, SPE-1, SPE-2, SPE-3, SPE-4 and ABQ-4 methods. The molecular feature 3-(2-hydroxyphenyl) propanoate, 12,13-EpOME and 12,13-DiHOME were present in multiple extractions.

Solid-phase extraction method resulted on a total of 677 molecular features (35.97% of total) and 174 exclusive molecular features (9.25% of total). The SPE had 48 annotated molecular features. N-[2-(1H-Indol-3-yl)ethyl]acetamide, 9-octadecenamide, EL and 7b,9-dihydroxy-3-(hydroxymethyl)-1,1,6,8-tetramethyl-5-oxo-1,1a,1b,4,4a,5,7a,7b,8,9-decahydro-9aH-cyclopropa[3,4]benzo[1,2-e]azulen-9a-yl acetate were observed for all SPE methods. 5-aminopentanoic acid, nicotinic acid, methionine, xanthine, azelaic acid and other 10 molecular features were observed in the SPE-B aliquots, which are usually discarded when SPE is used (Supplementary Table S3).

The original QuEChERS extraction method resulted on the extraction of 531 molecular features (24.92% of total) and had the lowest abundance of exclusive molecular features (38; 2.02% of total). A total of 34 molecular features extracted using the original QuEChERS were annotated. Acid-base QuEChERS and buffered QuEChERS extracted a total of 707 and 531 molecular features, respectively. This corresponded to 37.57 and 28.21% of total observed molecular features, respectively. Acid-base QuEChERS and buffered QuEChERS had more exclusive molecular features compared to original QuEChERS, 109 (5.79% of total) and 177 (9.40% of total), respectively. A total of 50 and 45 identified molecular features were extracted by the acid-base QuEChERS and buffered QuEChERS method, respectively.

### Mass spectrometry-based Molecular Networking of the rumen fluid

**Figure 2:**
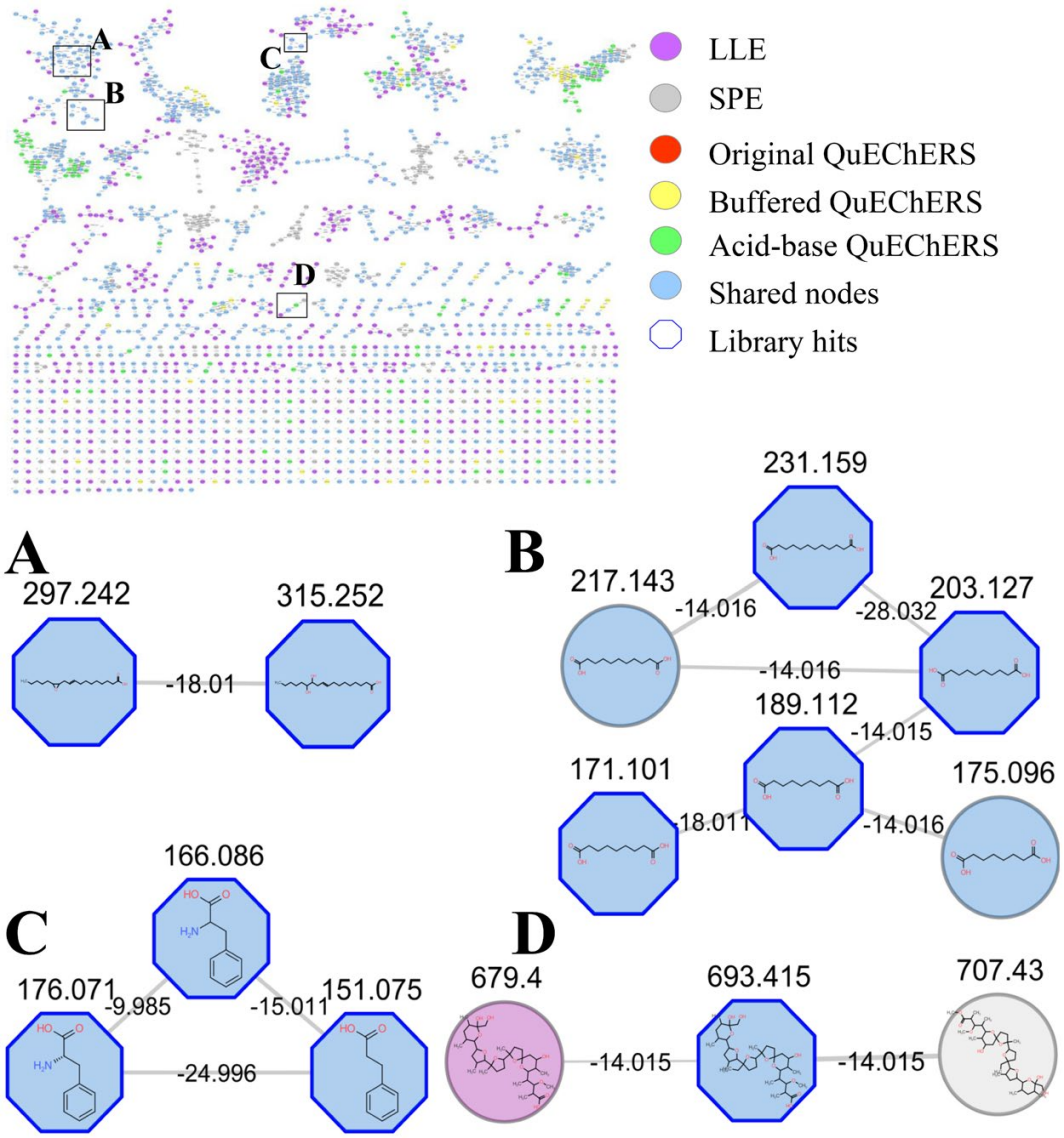
Molecular network (MN) generated using molecular features from the rumen fluid of a non-lactating dairy cow extracted using liquid-liquid extraction (LLE); solid-phase extraction (SPE); original, buffered and acid-base QuEChERS and analyzed using ultra-high performance liquid chromatography coupled to a high resolution mass spectrometer in tandem. Ellipse shaped nodes with gray border indicate putative assignments and octagon nodes with blue border indicate hits with Global Natural Products Social (GNPS). Purple, gray, red, yellow and greed nodes represent molecular features extracted exclusively with LLE, SPE, original, buffered and acid-base QuEChERs, respectively. Blue nodes represent molecular features extracted by more than one extraction method. *m/z* of mass shift between the nodes are displayed over the edges. (**A**) Octagon nodes with blue border indicate spectral match of the GNPS with 12,13-EpOME ([M+H]^+^; *m/z* 297.2430) and 12,13-DiHOME ([M+H]^+^; *m/z* 315.2534). (**B**) Octagon nodes with blue border indicate spectral match of the GNPS with azelaic acid ([M+H]^+^; *m/z* 189.112 and [M-H2O+H]^+^; *m/z* 171.101), decanedioic acid ([M+H]^+^; *m/z* 203.127) and dodecanedioic acid ([M+H]^+^; *m/z* 231.159); ellipse nodes with gray border indicate putative assignments to undecanedioic acid ([M+H]^+^; *m/z* 217.143) and suberic acid ([M+H]^+^; *m/z* 175.096). (**C**) Octagon nodes with blue border indicate spectral match of the GNPS with phenylalanine ([M+H]^+^; *m/z* 166.086), 3-indoleacetic acid ([M+H]^+^; *m/z* 176.071) and hydrocinnamic acid ([M+H]^+^; *m/z* 151.075). (**D**) Octagon node with blue border indicate spectral match of the GNPS with monensin ([M +Na]^+^; *m/z* 693.415) and ellipse nodes indicate its analogues monensin B ([M+Na]^+^; *m/z* 679.402) extracted exclusively on LLE, and monensin methyl ester ([M+Na]^+^; *m/z* 707.433) extracted exclusively on SPE.

The five extraction methods resulted on a diversity of annotated compounds, such as amino acids, dicarboxylic acids, carboxylic acids, lactones, lignans, fatty acids derivatives and indole compounds. Molecular features were visualized using the GNPS (Fig. 2). Four clusters with identified compounds are presented. Cluster A (Fig. 2A) was formed by 12,13-EpOME (*m/z* 297.243) and 12,13-DiHOME (*m/z* 315.253). Cluster B (Fig. 2B) was formed by dicarboxylic acids: azelaic acid ([M + H]^+^; *m/z* 189.112 and [M-H_2_O + H]^+^; *m/z* 171.101), decanedioic acid ([M + H]^+^; *m/z* 203.127) and dodecanedioic acid ([M + H]^+^; *m/z* 231.159). Cluster C was formed by phenylalanine ([M + H]^+^; *m/z* 166.086), 3-indoleacetic acid ([M + H]^+^; *m/z* 176.071) and hydrocinnamic acid [M + H]^+^; (*m/z* 151.075) (Fig. 2C). Cluster D was formed by monensin ([M + Na]^+^; *m/z* 693.415), monensin B ([M + Na]^+^; *m/z* 679.402) and monensin methyl ester ([M + Na]^+^; *m/z* 707.433; Fig. 2D).

## Discussion

The extraordinary activity of microorganisms is based on their remarkable metabolic diversity and genetic adaptability, which makes them an important source of genetic resources for biotechnological advancement and sustainable development. The success for biotechnological processes is directly related to the diversity of microorganisms and the molecules they produce as a result of primary and secondary metabolism as well as the conservation of the genetic resources they provide. The rumen fluid is a complex matrix composed by a myriad of microorganisms, which interact among themselves and the host, and degrade plant material (i.e. cellulose, hemicellulose, lignin, starch, protein and small amount of oil) from various sources. The intra- and interspecies interaction are responsible for the synergistic effect on the production of volatile fatty acids and microbial protein in the rumen. Despite the ability of the ruminant to degrade cellulose being responsibility of the microbiota, only recently a reference microbial genome catalog of the rumen was published, and yet a large portion of microorganisms remains unknown^16^. Furthermore, many of the rumen bacteria remain uncultured and uncharacterized, which is more troublesome for eukaryotes^17^. Thus, the use of LC-MS favors the exploration of the interactome, rather than the metagenome. This is actually an important feature as integrative omics tools are only becoming available. For example, the exploration of the metabolome can result on the discovery of novel compounds, such as antimicrobials^18^ or other bioactive molecules^19^. To date, the majority of rumen metabolome studies have used UHPLC-MS, gas chromatography-mass spectrometry and nuclear magnetic resonance, with the latter the most used due to reliability and absolute quantification^20^. However, UHPLC-MS has the advantage of having increased sensitivity and coverage. Thus, a single UHPLC-MS run can result on huge amount of data. For example, we obtained 432,391 MS spectra in this study. To overcome the difficulty to analyze this massive amount of data, the GNPS MN was used to comprehensively visualize the dataset and to prospect novel compounds. Another challenge of metabolomics studies relies on our ability to annotate unknown molecular features or their origins. Indeed, there is evidence that only 1.8% of spectra in untargeted metabolomics experiment can be annotated^21^. To overcome this issue, metabolites spectra can be compared based on parent and fragment ion similarities to publicly available molecular matches.

Recent studies exploring the rumen fluid metabolome have mainly focused on how the metabolites are influenced by different diets. For example, organic acids, amino acids, amines, sugars and nucleosides/nucleotides, which are the core of the rumen fluid, are affected when dairy cows are fed diets varying in levels of concentrate^22^. Using targeted approaches and a combination of analytical methods on the rumen fluid of cows fed diets varying in levels of barley, Saleem *et al*.^8^ observed 246 metabolites including: phospholipids, inorganic ions, gases, amino acids, dicarboxylic acids, fatty acids, volatile fatty acids, glycerides, carbohydrates and cholesterol esters. Aiming to identify markers for the rumen function that could lead to differences in average daily gain, Artegoitia^9^ observed 1,429 molecular features using the metabolomics untargeted approach. However, a large number of animals was used in that study (n = 16).

In our study, 1,882 molecular features were observed and 67 were identified, which was 3.56% of total. The sources of these uncharacterized molecular features may range from secreted molecules by ruminal microorganisms, plants, the host, and administered drugs. This offers a possibility to further explore the ruminant in its environment. For example, bacterial secondary metabolites can be used to identify antimicrobial resistance^23^. Moreover, due to the vast microbial interaction and dispute inside the rumen, the rumen is a pool for novel antimicrobials^24^. Furthermore, the GNPS MN is a collaborative platform that is improved from the spectra deposited by users. The rumen is still a poorly explored environment and deposit of rumen metabolome dataset is required.

Liquid-liquid extraction method resulted on the greatest abundance of molecular features: a total of 61.96%, and it also resulted on the greatest quantity of annotated compounds. Liquid-liquid extraction method extracts compounds with moderate polarity, and nonpolar or hydrophobic characteristics. The pH of the extraction can also be explored as it influences the solubility of acidic and basic compounds. Thus, pH modifiers can increase the abundance of dissociated compounds in the organic phase^25,26^. Alternatively, SPE methods are focused on low to medium polarity compounds using octadecylsilane (C18) and Cyano (CN) phase cartridges, respectively. To increase the abundance of extracted compounds, solvents with different elution strengths can be used. Lastly, QuEChERS was used as alternative extraction method for compounds of medium and low polarity, but with lower material and labor cost compared to SPE. QuEChERS extraction methods is based on an extraction step that partition with organic solvent, followed by a clean-up step with sorbents, which improves the selectivity to the method.

The molecular feature 3-(2-hydroxyphenyl) propanoic acid ([M]^−^; *m/z* 165.056), which was present in multiple extractions is metabolized from cinnamic acid^27^ and is produced by the anaerobic bacterium *Clostridium xylanolyticum*^28^. Another annotated compound was the EL, which has potential to improve milk stability, the immune system of the dairy cow, and to prevent cardiovascular diseases, osteoporosis and diabetes in human^29,30^.

Phenylalanine ([M + H]^+^; *m/z* 166.086), 3-indoleacetic acid ([M + H]^+^; *m/z* 176.071) and hydrocinnamic acid [M + H]^+^; (*m/z* 151.075) formed a cluster (Fig. 2C), being the latter produced by lactic acid bacteria^31,32^. Two oxylipins, 12,13-EpOME (*m/z* 297.243) and 12,13-DiHOME (*m/z* 315.253) had a match with the GNPS, which were also identified by Artegoitia^9^. These are bioactive lipids derived from several polyunsaturated fatty acids, such as linoleic acid. The epoxidation metabolites of linoleic acid produce the epoxyoctadecenoic acid (12,13-EpOME) and the epoxide hydration product dihydroxyoctadecenoic acid, (12,13-DiHOME). The modulation of these products can be associated with processes of inflammation in dairy cattle^33,34^.

One important feature of using the MN is the possibility to visualize structural similarities between molecules and the connection based similarities of their corresponding MS/MS fragmentation patterns. Therefore, the assertive annotation of a compound in the MN can implicate in the identification of neighboring nodes, which allows a more in-depth annotation and discovery of novel compounds. Indeed, we were able to explore the MN using this approach.

Monensin was observed and had the *m/z* of 693.415 ([M + Na]^+^). By observing the molecular features in the monensin cluster and the fragmentation pattern, we could infer that the unannotated structures *m/z* 679.402 and 707.433 are molecules structurally similar to monensin. Mass differences of −14 Da and +14 Da with monensin suggested differences in a methyl group in both cases. Inspecting the fragmentation pattern and comparing to public available data suggests both molecular features are monensin B ([M + Na]^+^; *m/z* 679.402) and monensin methyl ester ([M + Na]^+^; *m/z* 707.433)^35,36^.

Another example is related to azelaic acid, decanedioic acid ([M + H]^+^; *m/z* 203.127) and dodecanedioic acid ([M + H]^+^; *m/z* 231.159), which formed a cluster in the MN. These molecular features were observed the rumen of both cows and sheep and can be derived from the microbial hydrogenation of non-conjugated dienoic acids^37^. Azelaic acid ([M + H]^+^; *m/z* 189.112 and [M-H_2_O + H]^+^; *m/z* 171.101) is an organic compound observed in wheat, rice, rye and barley^38^ and can be used as carbon sources by the rumen microbiota. The proximity of the unannotated structure *m/z* 217.143 to decanedioic acid and dodecanedioic acid nodes, and the evaluation of the fragmentation pattern supported that the unannotated structure *m/z* 217.143 belonged to the dicarboxylic acids family. Mass differences of −14 Da with decanedioic and dodecanedioic acids suggested differences of CH_2_ group in both cases. The fragmentation pattern compared to public available data suggested the structure of undecanedioic acid ([M + H]^+^; 217.143). This characteristic mass difference was also observed between the unannotated structure *m/z* 175.096 and the azelaic acid. The loss of -14 Da and fragmentation pattern suggested that the molecule was the suberic acid ([M + H]^+^; 175.096).

## Conclusions

This investigation of the rumen fluid metabolome of a non-lactating Holstein cow using different extraction methods, liquid chromatography-mass spectrometry and Molecular Networking analysis resulted on 1,882 observed molecular features, which is the greatest abundance of molecular features reported to date. Several extraction methods resulted on various quantities of exclusive molecular features. The liquid-liquid extraction method had more exclusive and total molecular features. Only 67 molecular features observed had positive match with the Global Natural Products Social Molecular Networking database and public libraries, which corresponded to 3.56% total molecular features. Of those, amino acids, dicarboxylic acids, carboxylic acids, lactones, lignans, fatty acids derivatives and indole compounds were observed. Four clusters observed in the Molecular Networking were presented and two clusters were manually explored so that novel compounds could be identified. This highlights the use of ultra-high performance liquid chromatography tandem mass-spectrometry and Molecular Networking to explore the potential of the rumen as a reservoir for novel compounds.

## Electronic supplementary material

Supplementary Material

## Data Availability

The data set has been submitted to Global Natural Products Social Molecular Networking (GNPS) and are available via [https://gnps.ucsd.edu/ProteoSAFe/status.jsp?task=ab6ca77deedc4c65bc71b3043608c415] study identifier [171122_LRC4_attributesok].
