# Supplementary material for: Exploring the rumen fluid metabolome using liquid chromatography-high-resolution mass spectrometry and Molecular Networking
Source: Sci Rep. 2018 Dec 19;8:17971. doi: 10.1038/s41598-018-36196-4 (PMC6299289; doi:10.1038/s41598-018-36196-4)
Supplement: Supplementary file 1 — Supplementary Material [file 41598_2018_36196_MOESM1_ESM.docx]

***Supplementary Material***

Exploring the rumen fluid metabolome using liquid chromatography-high-resolution mass spectrometry and molecular networking

Rafaela Takako Ribeiro de Almeida^1^, Rodolpho Martin do Prado^1,2^, Carla Porto^1^, Geraldo Tadeu dos Santos^2,^ Sharon Huws^3^, Eduardo Jorge Pilau^1,*^

^1^LaBioMass, Chemistry Department, Universidade Estadual de Maringá, Maringá, Brazil

^2^Animal Science Department, Universidade Estadual de Maringá, Maringá, Brazil

^3^Institute for Global Food Security, Queen’s University of Belfast, Northern Ireland, UK

^*^Correspondence

Dr. Eduardo Jorge Pilau

e-mail address: [ejpilau@uem.br](mailto:ejpilau@uem.br)

Telephone number: +55 44 3011-5098

**Method S1**

**Generation of a Molecular Network using the Global Natural Products Social Molecular Networking**

Molecular Networking approach required the conversion of mass spectrometry raw data into mzXML file format followed by upload to the Global Natural Products Social Molecular Networking (GNPS). The MN was generated so that edges had a cosine score above 0.65 and 4 matched peaks. Edges between two nodes were kept in the MN if each of the nodes appeared in each other's respective top 10 most similar nodes. The data was filtered using all MS/MS peaks within +/- 17 Da of the precursor m/z, by removing them, and then clustering with MS-Cluster with parent mass and MS/MS fragment ion tolerances of 0.02 Da. Consensus spectra that contained less than 2 spectra were discarded. The spectra in the MN were then searched against GNPS spectral libraries. The library spectra were filtered in the same manner as the input data. All matches between network spectra and library spectra were required to have a score above 0.65 and at least 4 matched peaks. The MN data was downloaded and visualized in Cytoscape. Network fragmentation spectra in agreement with libraries spectra were manually confronted with fragmentation spectra of the proposed compounds and errors were calculated.

**Table S1**

**Identification, molecular formula, monoisotopic mass, observed *m/z* of ion and error of metabolites from the rumen fluid of a non-lactating Holstein cow**

| Compound number | | Metabolite | Formula | Monoisotopic mass | *m/z* _Exp_ positive | *m/z* _Exp_ negative | Error (ppm) |
| --- | --- | --- | --- | --- | --- | --- | --- |
| 01 | | Histamine | C_5_H_9_N_3_ | 111.0796 | 112.0867 | - | 6.89 |
| 02 | | Delta-Hexanolactone | C_6_H_10_O_2_ | 114.0681 | 115.0749 | - | 8.73 |
| 03 | | 5-Aminopentanoic acid | C_5_H_11_NO_2_ | 117.0790 | 118.0870 | - | -1.66 |
| 04 | | Phenylethylamine | C_8_H_11_N | 121.0891 | 122.0965 | - | 3.88 |
| 05 | | Nicotinic acid | C_6_H_5_NO_2_ | 123.0320 | 124.0392 | - | 5.26 |
| 06 | | Phloroglucinol | C_6_H_6_O_3_ | 126.0317 | 127.0384 | - | 8.81 |
| 07 | | Leucine | C_6_H_13_NO_2_ | 131.0946 | 132.1018 | - | 4.95 |
| 08 | | Cinnamaldehyde | C_9_H_8_O | 132.0575 | 133.0650 | - | 2.56 |
| 09 | | Oxindole | C_8_H_7_NO | 133.0528 | 134.0596 | - | 7.38 |
| 10 | | Alpha-Pinene-oxide^a^ | C_10_H_16_O | 152.1201 | 135.1170 | - | -1.28 |
| 11 | | Adenine | C_5_H_5_N_5_ | 135.0545 | 136.0615 | - | 6.03 |
| 12 | | Phenacylamine | C_8_H_9_N_O_ | 135.0684 | 136.0755 | - | 5.43 |
| Continued |  | | | | | | |
| Compound number | | Metabolite | Formula | Monoisotopic mass | *m/z* _Exp_ positive | *m/z* _Exp_ negative | Error (ppm) |
| 13 | | Phenylacetid acid | C_8_H_8_O_2_ | 136.0524 | 137.0594 | - | 6.23 |
| 14 | | Tyramine | C_8_H_11_NO | 137.0841 | 138.0912 | - | 4.99 |
| 15 | | Urocanic acid | C_6_H_6_N_2_O_2_ | 138.0429 | 139.0499 | - | 6.13 |
| 16 | | Methionine | C_5_H_11_NO_2_S | 149.0511 | 150.0581 | - | 5.16 |
| 17 | | N-(2-methylphenyl)acetamide | C_9_H_11_NO | 149.0841 | 150.0914 | - | 3.26 |
| 18 | | Hydrocinnamic acid | C_9_H_10_O_2_ | 150.0681 | 151.0751 | - | 5.33 |
| 19 | | Guanine | C_5_H_5_N_5_O | 151.0494 | 152.0562 | - | 6.81 |
| 20 | | Xanthine | C_5_H_4_N_4_O_2_ | 152.0334 | 153.0410 | - | 1.63 |
| 21 | | Limonene-1,2-oxide | C_10_H_16_O | 152.1201 | 153.1277 | - | 1.57 |
| 22 | | N-Acetylhistamine | C_7_H_11_N_3_O | 153.0902 | 154.0974 | - | 4.13 |
| 23 | | 3-Methyladipic acid | C_7_H_12_O_4_ | 160.0736 | 161.0812 | - | 1.14 |
| 24 | | 1H-Indole-3-carboxylic acid | C_9_H_7_NO_2_ | 161.0477 | 162.0549 | - | 3.72 |
| 25 | | Indole-3-ethanol | C_10_H_11_NO | 161.0841 | 162.0917 | - | 1.17 |
| 26 | | N-(2-Phenylethyl) acetamide | C_10_H_13_NO | 163.0997 | 164.1070 | - | 3.28 |
| Continued | |  |  |  |  |  |  |
| Compound number | | Metabolite | Formula | Monoisotopic mass | *m/z* _Exp_ positive | *m/z* _Exp_ negative | Error (ppm) |
| 27 | | 3-(2-hydroxyphenyl) propanoate | C_9_H_9_O_3_ | 165.0552 | - | 165.0558 | -3.82 |
| 28 | | Phenylalanine | C_9_H_11_NO_2_ | 165.0790 | 166.0866 | - | 1.23 |
| 29 | | Isovanillic acid | C_8_H_8_O_4_ | 168.0423 | 169.0494 | - | 4.05 |
| 30 | | Diphenylamine | C_12_H_11_N | 169.0891 | 170.0970 | - | -0.15 |
| 31 | | Suberic acid | C_8_H_14_O_4_ | 174.0892 | 175.0972 | - | -0.95 |
| 32 | | 3-Indoleacetic acid | C_10_H_9_NO_2_ | 175.0633 | 176.0711 | - | 0.31 |
| 33 | | 4-(2,6,6-Trimethyl-1-cyclohexen-1-yl)-2-butanone^a^ | C_13_H_22_O | 194.1671 | 177.1637 | - | 0.43 |
| 34 | | N-Acetyltyramine | C_10_H_13_NO_2_ | 179.0946 | 180.1017 | - | 4.19 |
| 35 | | Dihydroactinidiolide | C_11_H_16_O_2_ | 180.1150 | 181.1227 | - | 0.86 |
| 36 | | Tyrosine | C_9_H_11_NO_3_ | 181.0739 | 182.0810 | - | 3.94 |
| 37 | | 4-Pyridoxic acid | C_8_H_9_NO_4_ | 183.0532 | 184.0601 | - | 4.80 |
| 38 | | Delta-Undecalactone | C_11_H_20_O_2_ | 184.1463 | 185.1537 | - | 2.46 |
| 39 | | 4-(Phenylamino)phenol | C_12_H_11_NO | 185.0841 | 186.0917 | - | 1.02 |
| 40 | | Azelaic acid | C_9_H_16_O_4_ | 188.1049 | 189.1119 | - | 4.15 |
| Continued | |  |  |  |  |  |  |
| Compound number | | Metabolite | Formula | Monoisotopic mass | *m/z* _Exp_ positive | *m/z* _Exp_ negative | Error (ppm) |
| 41 | | Cyclo-(L-pro-L-val) | C_10_H_16_N_2_O_2_ | 196.1212 | 197.1279 | - | 5.60 |
| 42 | | Syringic acid | C_9_H_10_O_5_ | 198.0528 | 199.0595 | - | 5.77 |
| 43 | | Gamma-Dodecalactone | C_12_H_22_O_2_ | 198.1620 | 199.1698 | - | 0.03 |
| 44 | | N-[2-(1H-Indol-3-yl)ethyl]acetamide | ‎C_12_H_14_N_2_O | 202.1106 | 203.1179 | - | 2.65 |
| 45 | | Decanedioic acid | C_10_H_18_O_4_ | 202.1205 | 203.1276 | - | 3.61 |
| 46 | | Tryptophan | C_11_H_12_N_2_O_2_ | 204.0899 | 205.0967 | - | 4.89 |
| 47 | | 1,11-Undecanedioic acid | C_11_H_20_O_4_ | 216.1362 | 217.1433 | - | 3.15 |
| 48 | | Acetamiprid | C_10_H_11_ClN_4_ | 222.0672 | 223.0754 | - | -1.57 |
| 49 | | Dodecanedioic acid | C_12_H_22_O_4_ | 230.1518 | 231.1592 | - | 1.88 |
| 50 | | Costunolide | C_15_H_20_O_2_ | 232.1463 | 233.1540 | - | 0.66 |
| 51 | | 1,11-Undecanedicarboxylic acid | C_13_H_24_O_4_ | 244.1675 | 245.1745 | - | 3.20 |
| 52 | | Daidzein | C_15_H_10_O_4_ | 254.0579 | 255.0652 | - | 2.09 |
| 53 | | Tetradecanedioic acid | C_14_H_26_O_4_ | 258.1831 | - | 257.1756 | -1.23 |
| 54 | | Triphenylphosphine oxid | C_18_H_15_OP | 278.0861 | 279.0939 | - | -0.08 |
| Continued | |  |  |  |  |  |  |
| Compound number | | Metabolite | Formula | Monoisotopic mass | *m/z* _Exp_ positive | *m/z* _Exp_ negative | Error (ppm) |
| 55 | | Pinolenic acid | C_18_H_30_O_2_ | 278.2246 | 279.2316 | - | 2.88 |
| 56 | | 9-Octadecenamide | C_18_H_35_NO | 281.2719 | 282.2792 | - | 1.74 |
| 57 | | OPDA | C_18_H_28_O_3_ | 292.2038 | 293.2115 | - | 0.58 |
| 58 | | 12,13-EpOME | C_18_H_32_O_3_ | 296.2351 | 297.2430 | - | -0.10 |
| 59 | | Enterolactone | C_18_H_18_O_4_ | 298.1205 | 299.1278 | - | 1.79 |
| 60 | | 12,13-DiHOME | C_18_H_34_O_4_ | 314.2457 | 315.2534 | - | 0.43 |
| 61 | | Glycerol 1-hexadecanoate | C_19_H_38_O_4_ | 330.2770 | 331.2844 | - | 1.31 |
| 62 | | 13,14-Dihydro Prostaglandin F1a | C_20_H_38_O_5_ | 358.2719 | 359.2803 | - | -1.53 |
| 63 | | Niranthin | C_24_H_32_O_7_ | 432.2148 | 415.2140 | - | -5.98 |
| 64 | | 7b,9-Dihydroxy-3-(hydroxymethyl)-1,1,6,8-tetramethyl-5-oxo-1,1a,1b,4,4a,5,7a,7b,8,9-decahydro-9aH-cyclopropa[3,4]benzo[1,2-e]azulen-9a-yl acetate^b^ | C_22_H_30_O_6_ | 390.2042 | 432.2388 | - | -1.70 |
| 65 | | Monensin B^c^ | C_35_H_60_O_11_ | 656.4136 | 679.4027 | - | 0.93 |
| 66 | | Monensin^c^ | C_36_H_62_O_11_ | 670.4292 | 693.4173 | - | 2.42 |
| 67 | | Monensin methyl ester^c^ | C_37_H_64_O_11_ | 684.4449 | 707.4334 | - | 1.74 |

^a^Ion observed with H_2_O loss [M-H_2_O+H]^+^, ^b^Ion observed with CH_3_CN aduct [M+CH_3_CN+H]^+^, ^c^Ion observed with sodium aduct [M+Na]^+^.

**Supplementary Table S2**

**Identification and International Union of Pure and Applied Chemistry (IUPAC) name of metabolites from the rumen fluid of a non-lactating Holstein cow**

| Compound number | Metabolite | IUPAC Name |
| --- | --- | --- |
| 01 | Histamine | 2-(1H-Imidazol-4-yl)ethan-1-amine |
| 02 | Delta-Hexanolactone | 6-Methyloxan-2-one |
| 03 | 5-Aminopentanoic acid | 5-Aminopentanoic acid |
| 04 | Phenylethylamine | 2-Phenylethan-1-amine |
| 05 | Nicotinic acid | Pyridine-3-carboxylic acid |
| 06 | Phloroglucinol | Benzene-1,3,5-triol |
| 07 | Leucine | (2S)-2-Amino-4-methylpentanoic acid |
| 08 | Cinnamaldehyde | (2E)-3-Phenylprop-2-enal |
| 09 | Oxindole | 3H-Indol-2-ol |
| 10 | Alpha-Pinene-oxide ** | 2,7,7-Trimethyl-3-oxatricyclo[4.1.1.0²,⁴]octane |
| 11 | Adenine | 7H-Purin-6-amine |
| 12 | Phenacylamine | 2-Amino-1-phenylethan-1-one |
| Continued |  |  |
| Compound number | Metabolite | IUPAC Name |
| 13 | Phenylacetid acid | 2-Phenylacetic acid |
| 14 | Tyramine | 4-(2-Aminoethyl)phenol |
| 15 | Urocanic acid | (2E)-3-(1H-Imidazol-4-yl)prop-2-enoic acid |
| 16 | Methionine | (2S)-2-Aamino-4-(methylsulfanyl)butanoic acid |
| 17 | N-(2-Methylphenyl)acetamide | N-(2-Methylphenyl)acetamide |
| 18 | Hydrocinnamic acid | 3-Phenylpropanoic acid |
| 19 | Guanine | 2-Amino-6,7-dihydro-3H-purin-6-one |
| 20 | Xanthine | 2,3,6,7-Tetrahydro-1H-purine-2,6-dione |
| 21 | Limonene-1,2-oxide | 1-Methyl-4-(prop-1-en-2-yl)-7-oxabicyclo[4.1.0]heptane |
| 22 | N-Acetylhistamine | N-[2-(1H-Imidazol-5-yl)ethyl]acetamide |
| 23 | 3-Methyladipic acid | (3S)-3-Methylhexanedioic acid |
| 24 | 1H-Indole-3-carboxylic acid | 1H-Indole-3-carboxylic acid |
| 25 | Indole-3-ethanol | 2-(1H-Indol-3-yl)ethan-1-ol |
| 26 | N-(2-Phenylethyl) acetamide | N-(2-Phenylethyl)acetamide |
| Continued |  |  |
| Compound number | Metabolite | IUPAC Name |
| 27 | 3-(2-Hydroxyphenyl) propanoate | 3-(2-Hydroxyphenyl)propanoate |
| 28 | Phenylalanine | (2S)-2-Amino-3-phenylpropanoic acid |
| 29 | Isovanillic acid | 3-Hydroxy-4-methoxybenzoic acid |
| 30 | Diphenylamine | N-Phenylaniline |
| 31 | Suberic acid | Octanedioic acid |
| 32 | 3-Indoleacetic acid | 2-(1H-Indol-3-yl)acetic acid |
| 33 | 4-(2,6,6-Trimethyl-1-cyclohexen-1-yl)-2-butanone | 4-(2,6,6-Trimethyl-1-cyclohexen-1-yl)-2-butanone |
| 34 | N-Acetyltyramine | N-[2-(4-Hydroxyphenyl)ethyl]acetamide |
| 35 | Dihydroactinidiolide | 4,4,7a-Trimethyl-2,4,5,6,7,7a-hexahydro-1-benzofuran-2-one |
| 36 | Tyrosine | (2S)-2-Amino-3-(4-hydroxyphenyl)propanoic acid |
| 37 | 4-Pyridoxic acid | 3-Hydroxy-5-(hydroxymethyl)-2-methylpyridine-4-carboxylic acid |
| 38 | Delta-Undecalactone | 6-Hexyloxan-2-one |
| 39 | 4-(Phenylamino)phenol | 4-(Phenylamino)phenol |
| 40 | Azelaic acid | Nonanedioic acid |
| Continued |  |  |
| Compound number | Metabolite | IUPAC Name |
| 41 | Cyclo-(L-pro-L-val) | (3S,8aS)-3-Isopropylhexahydropyrrolo[1,2-a]pyrazine-1,4-dione |
| 42 | Syringic acid | 4-Hydroxy-3,5-dimethoxybenzoic acid |
| 43 | Gamma-Dodecalactone | 5-Octyloxolan-2-one |
| 44 | N-[2-(1H-Indol-3-yl)ethyl]acetamide | ‎ N-[2-(1H-Indol-3-yl)ethyl]acetamide |
| 45 | Decanedioic acid | Decanedioic acid |
| 46 | Tryptophan | (2S)-2-Amino-3-(1H-indol-3-yl)propanoic acid |
| 47 | 1,11-Undecanedioic acid | Undecanedioic acid |
| 48 | Acetamiprid | N-[(6-Chloropyridin-3-yl)methyl]-N'-cyano-N-methylethanimidamide |
| 49 | Dodecanedioic acid | Dodecanedioic acid |
| 50 | Costunolide | 6,10-Dimethyl-3-methylidene-2H,3H,3aH,4H,5H,8H,9H,11aH-cyclodeca[b]furan-2-one |
| 51 | 1,11-Undecanedicarboxylic acid | Tridecanedioic acid |
| 52 | Daidzein | 7-Hydroxy-3-(4-hydroxyphenyl)-4H-chromen-4-one |
| 53 | Tetradecanedioic acid | Tetradecanedioic acid |
| Continued |  |  |
| Compound number | Metabolite | IUPAC Name |
| 54 | Triphenylphosphine oxid | Diphenylphosphorylbenzene |
| 55 | Pinolenic acid | (5Z,9Z,12Z)-Octadeca-5,9,12-trienoic acid |
| 56 | 9-Octadecenamide | (9Z)-Octadec-9-enamide |
| 57 | OPDA | 8-[(1S,5S)-4-Oxo-5-[(Z)-pent-2-enyl]cyclopent-2-en-1-yl]octanoic acid |
| 58 | 12,13-EpOME | (9Z)-11-(3-Pentyloxiran-2-yl)undec-9-enoic acid |
| 59 | Enterolactone | 3,4-bis[(3-Hydroxyphenyl)methyl]oxolan-2-one |
| 60 | 12,13-DiHOME | (9Z)-12,13-Dihydroxyoctadec-9-enoic acid |
| 61 | Glycerol 1-hexadecanoate | 2,3-Dihydroxypropyl hexadecanoate |
| 62 | 13,14-Dihydro Prostaglandin F1a | 7-((1R,2R,3R,5S)-3,5-dihydroxy-2-((S)-3-hydroxyoctyl)cyclopentyl)heptanoic acid |
| 63 | Niranthin | 6-[(2R,3R)-3-[(3,4-Dimethoxyphenyl)methyl]-4-methoxy-2-(methoxymethyl)butyl]-4-methoxy-1,3-benzodioxole |
| 64 | 7b,9-Dihydroxy-3-(hydroxymethyl)-1,1,6,8-tetramethyl-5-oxo-1,1a,1b,4,4a,5,7a,7b,8,9-decahydro-9aH-cyclopropa[3,4]benzo[1,2-e]azulen-9a-yl acetate | 7b,9-Dihydroxy-3-(hydroxymethyl)-1,1,6,8-tetramethyl-5-oxo-1,1a,1b,4,4a,5,7a,7b,8,9-decahydro-9aH-cyclopropa[3,4]benzo[1,2-e]azulen-9a-yl acetate |
|  |  |  |
| Continued |  |  |
| Compound number | Metabolite | IUPAC Name |
| 66 | Monensin | (2S,3R,4S)-4-[(2S,5R,7S,8R,9S)-2-[(2R,5S)-5-Ethyl-5-[(2R,3S,5R)-5-[(2S,3S,5R,6R)-6-hydroxy-6-(hydroxymethyl)-3,5-dimethyloxan-2-yl]-3-methyloxolan-2-yl]oxolan-2-yl]-7-hydroxy-2,8-dimethyl-1,10-dioxaspiro[4.5]decan-9-yl]-3-methoxy-2-methylpentanoic acid |
| 67 | Monensin methyl ester | (2S,3R,4S)-methyl 4-((2S,5R,7S,8R,9S)-2-((2S,2'R,3'S,5R,5'R)-2-ethyl-5'-((2S,3S,5R,6R)-6-hydroxy-6-(hydroxymethyl)-3,5-dimethyltetrahydro-2H-pyran-2-yl)-3'-methyloctahydro-[2,2'-bifuran]-5-yl)-9-hydroxy-2,8-dimethyl-1,6-dioxaspiro[4.5]decan-7-yl)-3-methoxy-2-methylpentanoate |

**Table S3**

**Identified metabolites profile from the rumen fluid of a non-lactating Holstein cow extracted using liquid-liquid extraction (LLE), solid phase extraction (SPE), original, buffered and acid-base QuEChERS methods and fractions**

|  |  | Extraction method and fractions | | | | | | | | | | | | | | | | | | | | | | |
| --- | --- | --- | --- | --- | --- | --- | --- | --- | --- | --- | --- | --- | --- | --- | --- | --- | --- | --- | --- | --- | --- | --- | --- | --- |
| Compound number | Metabolite | LLE | | | SPE | | | | | | | | Original QuEChERS | | | Buffered QuEChERS | | | | | Acid-base QuEChERS | | | |
|  |  | LLE-1 | LLE-2 | LLE-3 | SPE-1B | SPE-2B | SPE-3B | SPE-4B | SPE-1A | SPE-2A | SPE-3A | SPE-4A | OQ-1 | OQ-2 | OQ-3 | BQ-1 | BQ-2 | BQ-3 | BQ-4 | BQ-5 | ABQ-1 | ABQ-2 | ABQ-3 | ABQ-4 |
| 01 | Histamine |  |  | x |  |  |  |  |  |  |  | x |  |  |  |  |  | x |  |  |  | x |  |  |
| 02 | Delta-Hexanolactone |  | x |  | x | x | x | x |  | x |  |  |  |  |  | x |  |  |  | x | x |  |  |  |
| 03 | 5-Aminopentanoic acid |  |  |  | x | x | x | x |  |  |  |  |  |  |  |  |  |  |  |  | x | x |  |  |
| 04 | Phenylethylamine | x |  |  |  |  |  |  |  |  |  |  | x |  |  |  |  |  |  |  |  |  |  |  |
| 05 | Nicotinic acid |  | x |  | x | x | x | x |  |  |  |  |  |  |  |  |  |  |  |  | x |  |  |  |
| 06 | Phloroglucinol | x | x | x | x | x | x | x | x | x | x | x | x | x | x | x | x | x | x | x | x | x | x | x |
| 07 | Leucine |  |  |  | x | x | x | x |  | x | x |  | x |  |  | x |  |  | x | x | x | x |  |  |
| 08 | Cinnamaldehyde | x | x |  |  |  |  |  |  |  |  |  | x |  |  | x |  |  |  |  | x |  |  |  |
| 09 | Oxindole | x | x |  |  |  |  |  |  |  |  |  | x |  |  |  |  |  |  |  |  |  |  |  |
| 10 | Alpha-Pinene-oxide | x |  | x |  |  |  |  |  |  |  |  |  |  |  |  |  |  |  |  |  |  |  |  |
| 11 | Adenine | x | x | x |  |  |  |  | x | x |  |  |  | x | x |  | x | x |  |  |  |  | x | x |
| 12 | Phenacylamine | x | x | x |  |  |  |  | x | x | x |  | x |  |  | x |  |  |  |  | x | x | x | x |
| 13 | Phenylacetid acid | x | x |  |  |  | x | x |  | x |  |  | x | x | x | x |  | x | x | x | x | x | x | x |
| 14 | Tyramine |  |  | x |  |  |  |  | x | x |  | x | x | x | x |  | x | x |  |  |  |  | x |  |
| 15 | Urocanic acid |  | x |  |  |  |  |  |  |  |  |  |  |  |  |  |  |  |  |  |  |  |  |  |
| 16 | Methionine |  |  |  | x |  | x | x |  |  |  |  |  |  |  |  |  |  |  |  | x | x |  |  |
| 17 | N-(2-methylphenyl)acetamide | x |  | x |  |  |  |  |  |  |  |  |  |  |  |  |  |  |  |  |  |  |  |  |
| 18 | Hydrocinnamic acid | x | x |  |  |  |  |  |  |  |  |  | x |  |  | x |  |  |  |  | x | x |  | x |
| 19 | Guanine | x | x |  |  |  |  |  |  |  |  |  |  |  |  |  |  |  |  |  |  |  |  |  |
| 20 | Xanthine |  |  |  | x | x | x |  |  |  |  |  |  |  |  |  |  | x |  |  | x |  |  |  |
| 21 | Limonene-1,2-oxide | x | x |  |  |  | x | x |  | x |  |  | x |  |  | x |  | x |  |  | x |  |  |  |
| 22 | N-Acetylhistamine | x |  | x |  |  |  |  |  |  |  |  |  |  |  | x |  |  |  |  |  |  |  |  |
| 23 | 3-Methyladipic acid |  |  |  | x |  | x | x |  |  |  |  |  |  |  |  |  |  |  |  | x |  |  |  |
| Continued | |  |  |  |  |  |  |  |  |  |  |  |  |  |  |  |  |  |  |  |  |  |  |  |

|  |  | Extraction method and fractions | | | | | | | | | | | | | | | | | | | | | | |
| --- | --- | --- | --- | --- | --- | --- | --- | --- | --- | --- | --- | --- | --- | --- | --- | --- | --- | --- | --- | --- | --- | --- | --- | --- |
| Compound number | Metabolite | LLE | | | SPE | | | | | | | | Original QuEChERS | | | Buffered QuEChERS | | | | | Acid-base QuEChERS | | | |
|  |  | LLE-1 | LLE-2 | LLE-3 | SPE-1B | SPE-2B | SPE-3B | SPE-4B | SPE-1A | SPE-2A | SPE-3A | SPE-4A | OQ-1 | OQ-2 | OQ-3 | BQ-1 | BQ-2 | BQ-3 | BQ-4 | BQ-5 | ABQ-1 | ABQ-2 | ABQ-3 | ABQ-4 |
| 24 | 1H-Indole-3-carboxylic acid | x | x | x |  |  |  |  | x | x |  |  | x |  |  | x |  |  | x | x | x | x |  | x |
| 25 | Indole-3-ethanol | x |  | x |  |  |  |  |  |  |  | x |  |  |  |  |  |  |  |  |  |  | x |  |
| 26 | N-(2-Phenylethyl) acetamide | x |  |  |  |  |  |  |  |  |  |  |  |  |  |  |  |  |  |  |  |  |  |  |
| 27 | 3-(2-hydroxyphenyl) propanoate | x | x |  | x | x | x | x |  |  |  |  | x |  | x | x | x | x | x | x | x | x |  | x |
| 28 | Phenylalanine |  |  |  | x |  | x | x |  |  |  |  |  |  |  | x |  |  | x | x | x | x |  |  |
| 29 | Isovanillic acid |  | x |  | x | x | x | x |  |  |  |  | x |  |  | x |  |  | x | x | x | x |  |  |
| 30 | Diphenylamine | x | x | x |  |  |  |  |  |  |  |  |  |  |  |  |  |  |  |  |  |  |  |  |
| 31 | Suberic acid |  | x |  | x |  | x | x |  |  |  |  |  |  |  |  |  |  | x | x | x |  |  |  |
| 32 | 3-Indoleacetic acid | x | x |  |  |  |  |  |  |  |  |  | x |  |  | x |  |  |  |  | x |  |  |  |
| 33 | 4-(2,6,6-Trimethyl-1-cyclohexen-1-yl)-2-butanone | x |  | x |  |  |  |  |  |  |  |  |  |  |  |  |  |  |  |  |  |  |  |  |
| 34 | N-Acetyltyramine | x |  | x |  |  |  |  | x | x |  |  | x |  |  | x |  |  |  |  | x |  |  |  |
| 35 | Dihydroactinidiolide | x | x | x |  |  |  |  |  | x | x | x | x | x | x | x | x | x | x | x | x | x | x |  |
| 36 | Tyrosine |  |  |  | x | x | x | x |  |  |  |  |  |  |  |  |  |  |  |  | x | x |  |  |
| 37 | 4-Pyridoxic acid |  |  |  | x | x | x | x |  |  |  |  |  |  |  | x |  |  |  |  | x | x |  |  |
| 38 | Delta-Undecalactone |  | x |  |  |  |  |  |  |  |  |  |  |  |  | x |  |  |  |  | x |  |  |  |
| 39 | 4-(Phenylamino)phenol | x |  | x |  |  |  |  |  |  |  |  |  |  |  |  |  |  |  |  |  |  |  |  |
| 40 | Azelaic acid |  | x |  | x | x | x | x |  |  |  |  |  |  |  | x |  | x |  | x | x |  |  |  |
| 41 | Cyclo-(L-pro-L-val) | x |  | x |  |  |  |  |  |  |  |  |  |  |  | x |  |  |  |  |  |  |  |  |
| 42 | Syringic acid |  | x |  |  |  |  |  |  |  |  |  |  |  |  | x |  |  |  |  | x |  |  |  |
| 43 | Gamma-Dodecalactone | x |  | x |  |  |  |  |  |  |  |  | x |  |  | x |  |  |  |  |  |  |  |  |
| 44 | N-[2-(1H-Indol-3-yl)ethyl]acetamide | x |  | x |  |  |  |  | x | x | x | x | x |  |  |  |  |  |  |  | x |  |  |  |
| 45 | Decanedioic acid |  | x |  | x | x | x | x |  |  |  |  | x |  |  | x |  | x |  | x | x |  |  |  |
| 46 | Tryptophan |  |  |  |  |  | x | x | x | x |  |  |  |  |  | x |  |  |  |  | x | x |  |  |
| Continued | |  |  |  |  |  |  |  |  |  |  |  |  |  |  |  |  |  |  |  |  |  |  |  |
|  |  | Extraction method and fractions | | | | | | | | | | | | | | | | | | | | | | |
| Compound number | Metabolite | LLE | | | SPE | | | | | | | | Original QuEChERS | | | Buffered QuEChERS | | | | | Acid-base QuEChERS | | | |
|  |  | LLE-1 | LLE-2 | LLE-3 | SPE-1B | SPE-2B | SPE-3B | SPE-4B | SPE-1A | SPE-2A | SPE-3A | SPE-4A | OQ-1 | OQ-2 | OQ-3 | BQ-1 | BQ-2 | BQ-3 | BQ-4 | BQ-5 | ABQ-1 | ABQ-2 | ABQ-3 | ABQ-4 |
| 47 | 1,11-Undecanedioic acid |  | x |  |  |  | x | x |  |  |  |  | x |  |  | x |  |  |  |  | x |  |  |  |
| 48 | Acetamiprid |  |  |  |  |  |  |  |  |  |  |  | x |  |  |  |  |  |  |  |  |  |  |  |
| 49 | Dodecanedioic acid |  | x |  |  |  | x | x |  |  |  |  | x |  |  | x | x | x | x | x | x | x |  |  |
| 50 | Costunolide | x | x |  |  |  |  |  | x | x |  |  | x |  |  | x |  |  |  |  | x |  |  |  |
| 51 | 1,11-Undecanedicarboxylic acid |  | x |  |  | x | x | x | x | x |  |  | x |  | x | x | x | x | x | x | x | x |  |  |
| 52 | Daidzein | x | x |  |  |  |  |  | x | x |  |  | x |  |  | x |  |  |  |  | x |  | x |  |
| 53 | Tetradecanedioic acid | x | x |  |  |  |  | x | x | x |  |  | x |  | x | x | x | x |  |  | x | x |  |  |
| 54 | Triphenylphosphine oxid | x | x | x |  |  |  |  | x | x |  | x | x |  |  | x |  |  |  |  | x | x |  | x |
| 55 | Pinolenic acid | x | x | x |  |  |  |  | x | x |  |  | x |  |  | x |  |  |  |  | x |  |  |  |
| 56 | 9-Octadecenamide | x | x | x |  |  |  |  | x | x | x | x | x | x | x | x | x | x | x |  |  | x | x | x |
| 57 | OPDA | x |  |  |  |  | x |  | x | x |  |  | x |  |  | x |  |  | x |  | x | x |  |  |
| 58 | 12,13-EpOME | x | x | x |  |  |  |  | x | x |  |  | x |  |  | x |  |  |  |  | x |  |  | x |
| 59 | Enterolactone | x |  | x |  |  |  |  | x | x | x | x |  |  |  |  |  |  |  |  |  |  |  | x |
| 60 | 12,13-DiHOME | x | x | x |  |  |  |  | x | x |  |  | x |  |  | x |  |  |  |  | x |  |  | x |
| 61 | Glycerol 1-hexadecanoate | x | x |  |  |  |  |  | x | x |  |  | x |  |  | x |  |  |  |  |  | x | x | x |
| 62 | 13,14-Dihydro Prostaglandin F1a | x |  |  |  | x |  |  | x | x |  |  |  |  |  |  |  |  |  |  | x |  |  |  |
| 63 | Niranthin | x | x | x |  |  |  |  | x |  |  |  | x | x | x | x | x |  |  |  | x | x | x | x |
| 64 | 7b,9-Dihydroxy-3-(hydroxymethyl)-1,1,6,8-tetramethyl-5-oxo-1,1a,1b,4,4a,5,7a,7b,8,9-decahydro-9aH-cyclopropa[3,4]benzo[1,2-e]azulen-9a-yl acetate | x | x | x |  |  |  |  | x | x | x | x | x | x | x | x | x | x | x | x | x | x | x | x |
| 65 | Monensin B | x |  | x |  |  |  |  |  |  |  |  |  |  |  |  |  |  |  |  |  |  |  |  |
| 66 | Monensin | x | x | x |  |  |  |  | x | x |  | x | x |  |  | x |  |  |  |  |  |  |  | x |
| 67 | Monensin methyl Ester |  |  |  |  |  |  |  |  | x |  |  |  |  |  |  |  |  |  |  |  |  |  |  |

**Figure S1**

Liquid-liquid extraction method used on the rumen fluid of a non-lactating Holstein cow. Plain and dashed arrows represent procedures and final extracts, respectively

**Figure S2**

Solid phase extraction method used on the rumen fluid of a non-lactating Holstein cow. Plain and dashed arrows represent procedures direction and final extracts, respectively

**Figure S3**

Original QuEChERS method used on the rumen fluid of a non-lactating Holstein cow. Plain and dashed arrows represent procedures direction and final extracts, respectively

**Figure S4**

Buffered QuEChERS method used on the rumen fluid of a non-lactating Holstein cow. Plain and dashed arrows represent procedures direction and final extracts, respectively

**Figure S5**

Acid-base QuEChERS method used on the rumen fluid of a non-lactating Holstein cow. Plain and dashed arrows represent procedures direction and final extracts, respectively

**Figure S6**

Histamine identified in the rumen fluid of a non-lactating Holstein cow using ultra-high performance liquid chromatography–tandem mass-spectrometry and the Global Natural Products Social Molecular Networking. Ion was identified as [M+H]^+^ with 6.89 ppm mass error

**Figure S7**

Delta-Hexanolactone identified in the rumen fluid of a non-lactating Holstein cow using ultra-high performance liquid chromatography–tandem mass-spectrometry and the Global Natural Products Social Molecular Networking. Ion was identified as [M+H]^+^ with 8.73 ppm mass error

**Figure S8**

5-Aminopentanoic acid identified in the rumen fluid of a non-lactating Holstein cow using ultra-high performance liquid chromatography–tandem mass-spectrometry and the Global Natural Products Social Molecular Networking. Ion was identified as [M+H]^+^ with -1.66 ppm mass error

**Figure S9**

Phenylethylamine identified in the rumen fluid of a non-lactating Holstein cow using ultra-high performance liquid chromatography–tandem mass-spectrometry and the Global Natural Products Social Molecular Networking. Ion was identified as [M+H]^+^ with 3.88 ppm mass error

**Figure S10**

Nicotinic acid identified in the rumen fluid of a non-lactating Holstein cow using ultra-high performance liquid chromatography–tandem mass-spectrometry and the Global Natural Products Social Molecular Networking. Ion was identified as [M+H]^+^ with 5.26 ppm mass error

**Figure S11**

Phloroglucinol identified in the rumen fluid of a non-lactating Holstein cow using ultra-high performance liquid chromatography–tandem mass-spectrometry and the Global Natural Products Social Molecular Networking. Ion was identified as [M+H]^+^ with 8.81 ppm mass error

**Figure S12**

Leucine identified in the rumen fluid of a non-lactating Holstein cow using ultra-high performance liquid chromatography–tandem mass-spectrometry and the Global Natural Products Social Molecular Networking. Ion was identified as [M+H]^+^ with 4.95 ppm mass error

**Figure S13**

Cinnamaldehyde identified in the rumen fluid of a non-lactating Holstein cow using ultra-high performance liquid chromatography–tandem mass-spectrometry and the Global Natural Products Social Molecular Networking. Ion was identified as [M+H]^+^ with 2.56 ppm mass error

**Figure S14**

Oxindole identified in the rumen fluid of a non-lactating Holstein cow using ultra-high performance liquid chromatography–tandem mass-spectrometry and the Global Natural Products Social Molecular Networking. Ion was identified as [M+H]^+^ with 7.38 ppm mass error

**Figure S15**

Alpha-Pinene-oxide identified in the rumen fluid of a non-lactating Holstein cow using ultra-high performance liquid chromatography–tandem mass-spectrometry and the Global Natural Products Social Molecular Networking. Ion was identified as [M-H_2_O+H]^+^ with -1.28 ppm mass error

**Figure S16**

Adenine identified in the rumen fluid of a non-lactating Holstein cow using ultra-high performance liquid chromatography–tandem mass-spectrometry and the Global Natural Products Social Molecular Networking. Ion was identified as [M+H]^+^ with 6.03 ppm mass error

**Figure S17**

Phenacylamine identified in the rumen fluid of a non-lactating Holstein cow using ultra-high performance liquid chromatography–tandem mass-spectrometry and the Global Natural Products Social Molecular Networking. Ion was identified as [M+H]^+^ with 5.43 ppm mass error

**Figure S18**

Phenylacetid acid identified in the rumen fluid of a non-lactating Holstein cow using ultra-high performance liquid chromatography–tandem mass-spectrometry and the Global Natural Products Social Molecular Networking. Ion was identified as [M+H]^+^ with 6.23 ppm mass error

**Figure S19**

Tyramine identified in the rumen fluid of a non-lactating Holstein cow using ultra-high performance liquid chromatography–tandem mass-spectrometry and the Global Natural Products Social Molecular Networking. Ion was identified as [M+H]^+^ with 4.99 ppm mass error

**Figure S20**

Urocanic acid identified in the rumen fluid of a non-lactating Holstein cow using ultra-high performance liquid chromatography–tandem mass-spectrometry and the Global Natural Products Social Molecular Networking. Ion was identified as [M+H]^+^ with 6.13 ppm mass error

**Figure S21**

Methionine identified in the rumen fluid of a non-lactating Holstein cow using ultra-high performance liquid chromatography–tandem mass-spectrometry and the Global Natural Products Social Molecular Networking. Ion was identified as [M+H]^+^ with 5.16 ppm mass error

**Figure S22**

N-(2-methylphenyl)acetamide identified in the rumen fluid of a non-lactating Holstein cow using ultra-high performance liquid chromatography–tandem mass-spectrometry and the Global Natural Products Social Molecular Networking. Ion was identified as [M+H]^+^ with 3.26 ppm mass error

**Figure S23**

Hydrocinnamic acid identified in the rumen fluid of a non-lactating Holstein cow using ultra-high performance liquid chromatography–tandem mass-spectrometry and the Global Natural Products Social Molecular Networking. Ion was identified as [M+H]^+^ with 5.33 ppm mass error

**Figure S24**

Guanine identified in the rumen fluid of a non-lactating Holstein cow using ultra-high performance liquid chromatography–tandem mass-spectrometry and the Global Natural Products Social Molecular Networking. Ion was identified as [M+H]^+^ with 6.81 ppm mass error

**Figure S25**

Xanthine identified in the rumen fluid of a non-lactating Holstein cow using ultra-high performance liquid chromatography–tandem mass-spectrometry and the Global Natural Products Social Molecular Networking. Ion was identified as [M+H]^+^ with 1.63 ppm mass error

**Figure S26**

Limonene-1,2-oxide identified in the rumen fluid of a non-lactating Holstein cow using ultra-high performance liquid chromatography–tandem mass-spectrometry and the Global Natural Products Social Molecular Networking. Ion was identified as [M+H]^+^ with 1.57 ppm mass error

**Figure S27**

N-Acetylhistamine identified in the rumen fluid of a non-lactating Holstein cow using ultra-high performance liquid chromatography–tandem mass-spectrometry and the Global Natural Products Social Molecular Networking. Ion was identified as [M+H]^+^ with 4.13 ppm mass error

**Figure S28**

3-Methyladipic acid identified in the rumen fluid of a non-lactating Holstein cow using ultra-high performance liquid chromatography–tandem mass-spectrometry and the Global Natural Products Social Molecular Networking. Ion was identified as [M+H]^+^ with 1.14 ppm mass error

**Figure S29**

1H-Indole-3-carboxylic acid identified in the rumen fluid of a non-lactating Holstein cow using ultra-high performance liquid chromatography–tandem mass-spectrometry and the Global Natural Products Social Molecular Networking. Ion was identified as [M+H]^+^ with 3.72 ppm mass error

**Figure S30**

Indole-3-ethanol identified in the rumen fluid of a non-lactating Holstein cow using ultra-high performance liquid chromatography–tandem mass-spectrometry and the Global Natural Products Social Molecular Networking. Ion was identified as [M+H]^+^ with 1.17 ppm mass error

**Figure S31**

N-(2-Phenylethyl) acetamide identified in the rumen fluid of a non-lactating Holstein cow using ultra-high performance liquid chromatography–tandem mass-spectrometry and the Global Natural Products Social Molecular Networking. Ion was identified as [M+H]^+^ with 3.28 ppm mass error

**Figure S32**

3-(2-hydroxyphenyl) propanoate identified in the rumen fluid of a non-lactating Holstein cow using ultra-high performance liquid chromatography–tandem mass-spectrometry and the Global Natural Products Social Molecular Networking. Ion was identified as [M]^-^ with -382 ppm mass error

**Figure S33**

Phenylalanine identified in the rumen fluid of a non-lactating Holstein cow using ultra-high performance liquid chromatography–tandem mass-spectrometry and the Global Natural Products Social Molecular Networking. Ion was identified as [M+H]^+^ with 1.23 ppm mass error

**Figure S34**

Isovanillic acid identified in the rumen fluid of a non-lactating Holstein cow using ultra-high performance liquid chromatography–tandem mass-spectrometry and the Global Natural Products Social Molecular Networking. Ion was identified as [M+H]^+^ with 4.05 ppm mass error

**Figure S35**

Diphenylamine identified in the rumen fluid of a non-lactating Holstein cow using ultra-high performance liquid chromatography–tandem mass-spectrometry and the Global Natural Products Social Molecular Networking. Ion was identified as [M+H]^+^ with -0.15 ppm mass error

**Figure S36**

Suberic acid identified in the rumen fluid of a non-lactating Holstein cow using ultra-high performance liquid chromatography–tandem mass-spectrometry and the Global Natural Products Social Molecular Networking. Ion was identified as [M+H]^+^ with -0.95 ppm mass error

**Figure S37**

3-Indoleacetic acid identified in the rumen fluid of a non-lactating Holstein cow using ultra-high performance liquid chromatography–tandem mass-spectrometry and the Global Natural Products Social Molecular Networking. Ion was identified as [M+H]^+^ with 0.31 ppm mass error

**Figure S38**

4-(2,6,6-Trimethyl-1-cyclohexen-1-yl)-2-butanone identified in the rumen fluid of a non-lactating Holstein cow using ultra-high performance liquid chromatography–tandem mass-spectrometry and the Global Natural Products Social Molecular Networking. Ion was identified as [M-H_2_O+H]^+^ with 0.43 ppm mass error

**Figure S39**

N-Acetyltyramine identified in the rumen fluid of a non-lactating Holstein cow using ultra-high performance liquid chromatography–tandem mass-spectrometry and the Global Natural Products Social Molecular Networking. Ion was identified as [M+H]^+^ with 4.19 ppm mass error

**Figure S40**

Dihydroactinidiolide identified in the rumen fluid of a non-lactating Holstein cow using ultra-high performance liquid chromatography–tandem mass-spectrometry and the Global Natural Products Social Molecular Networking. Ion was identified as [M+H]^+^ with 0.86 ppm mass error

**Figure S41**

Tyrosine identified in the rumen fluid of a non-lactating Holstein cow using ultra-high performance liquid chromatography–tandem mass-spectrometry and the Global Natural Products Social Molecular Networking. Ion was identified as [M+H]^+^ with 3.94 ppm mass error

**Figure S42**

4-Pyridoxic acid identified in the rumen fluid of a non-lactating Holstein cow using ultra-high performance liquid chromatography–tandem mass-spectrometry and the Global Natural Products Social Molecular Networking. Ion was identified as [M+H]^+^ with 4.80 ppm mass error

**Figure S43**

Delta-Undecalactone identified in the rumen fluid of a non-lactating Holstein cow using ultra-high performance liquid chromatography–tandem mass-spectrometry and the Global Natural Products Social Molecular Networking. Ion was identified as [M+H]^+^ with 2.46 ppm mass error

**Figure S44**

4-(Phenylamino)phenol identified in the rumen fluid of a non-lactating Holstein cow using ultra-high performance liquid chromatography–tandem mass-spectrometry and the Global Natural Products Social Molecular Networking. Ion was identified as [M+H]^+^ with 1.02 ppm mass error

**Figure S45**

Azelaic acid identified in the rumen fluid of a non-lactating Holstein cow using ultra-high performance liquid chromatography–tandem mass-spectrometry and the Global Natural Products Social Molecular Networking. Ion was identified as [M+H]^+^ with 4.15 ppm mass error

**Figure S46**

Cyclo-(L-pro-L-val) identified in the rumen fluid of a non-lactating Holstein cow using ultra-high performance liquid chromatography–tandem mass-spectrometry and the Global Natural Products Social Molecular Networking. Ion was identified as [M+H]^+^ with 5.60 ppm mass error

**Figure S47**

Syringic acid identified in the rumen fluid of a non-lactating Holstein cow using ultra-high performance liquid chromatography–tandem mass-spectrometry and the Global Natural Products Social Molecular Networking. Ion was identified as [M+H]^+^ with 5.77 ppm mass error

**Figure S48**

Gamma-Dodecalactone identified in the rumen fluid of a non-lactating Holstein cow using ultra-high performance liquid chromatography–tandem mass-spectrometry and the Global Natural Products Social Molecular Networking. Ion was identified as [M+H]^+^ with 0.03 ppm mass error

**Figure S49**

N-[2-(1H-Indol-3-yl)ethyl]acetamide identified in the rumen fluid of a non-lactating Holstein cow using ultra-high performance liquid chromatography–tandem mass-spectrometry and the Global Natural Products Social Molecular Networking. Ion was identified as [M+H]^+^ with 2.65 ppm mass error

**Figure S50**

Decanedioic acid identified in the rumen fluid of a non-lactating Holstein cow using ultra-high performance liquid chromatography–tandem mass-spectrometry and the Global Natural Products Social Molecular Networking. Ion was identified as [M+H]^+^ with 3.61 ppm mass error

**Figure S51**

Tryptophan identified in the rumen fluid of a non-lactating Holstein cow using ultra-high performance liquid chromatography–tandem mass-spectrometry and the Global Natural Products Social Molecular Networking. Ion was identified as [M+H]^+^ with 4.89 ppm mass error

**Figure S52**

1,11-Undecanedioic acid identified in the rumen fluid of a non-lactating Holstein cow using ultra-high performance liquid chromatography–tandem mass-spectrometry and the Global Natural Products Social Molecular Networking. Ion was identified as [M+H]^+^ with 3.15 ppm mass error

**Figure S53**

Acetamiprid identified in the rumen fluid of a non-lactating Holstein cow using ultra-high performance liquid chromatography–tandem mass-spectrometry and the Global Natural Products Social Molecular Networking. Ion was identified as [M+H]^+^ with -1.57 ppm mass error

**Figure S54**

Dodecanedioic acid identified in the rumen fluid of a non-lactating Holstein cow using ultra-high performance liquid chromatography–tandem mass-spectrometry and the Global Natural Products Social Molecular Networking. Ion was identified as [M+H]^+^ with 1.88 ppm mass error

**Figure S55**

Costunolide identified in the rumen fluid of a non-lactating Holstein cow using ultra-high performance liquid chromatography–tandem mass-spectrometry and the Global Natural Products Social Molecular Networking. Ion was identified as [M+H]^+^ with 0.66 ppm mass error

**Figure S56**

1,11-Undecanedicarboxylic acid identified in the rumen fluid of a non-lactating Holstein cow using ultra-high performance liquid chromatography–tandem mass-spectrometry and the Global Natural Products Social Molecular Networking. Ion was identified as [M+H]^+^ with 3.20 ppm mass error

**Figure S57**

Daidzein identified in the rumen fluid of a non-lactating Holstein cow using ultra-high performance liquid chromatography–tandem mass-spectrometry and the Global Natural Products Social Molecular Networking. Ion was identified as [M+H]^+^ with 2.09 ppm mass error

**Figure S58**

Tetradecanedioic acid identified in the rumen fluid of a non-lactating Holstein cow using ultra-high performance liquid chromatography–tandem mass-spectrometry and the Global Natural Products Social Molecular Networking. Ion was identified as [M+H]^+^ with -1.23 ppm mass error

**Figure S59**

Triphenylphosphine oxid identified in the rumen fluid of a non-lactating Holstein cow using ultra-high performance liquid chromatography–tandem mass-spectrometry and the Global Natural Products Social Molecular Networking. Ion was identified as [M+H]^+^ with -0.08 ppm mass error

**Figure S60**

Pinolenic acid identified in the rumen fluid of a non-lactating Holstein cow using ultra-high performance liquid chromatography–tandem mass-spectrometry and the Global Natural Products Social Molecular Networking. Ion was identified as [M+H]^+^ with 2.88 ppm mass error

**Figure S61**

9-Octadecenamide identified in the rumen fluid of a non-lactating Holstein cow using ultra-high performance liquid chromatography–tandem mass-spectrometry and the Global Natural Products Social Molecular Networking. Ion was identified as [M+H]^+^ with 1.74 ppm mass error

**Figure S62**

OPDA identified in the rumen fluid of a non-lactating Holstein cow using ultra-high performance liquid chromatography–tandem mass-spectrometry and the Global Natural Products Social Molecular Networking. Ion was identified as [M+H]^+^ with 0.58 ppm mass error

**Figure S63**

12,13-EpOME identified in the rumen fluid of a non-lactating Holstein cow using ultra-high performance liquid chromatography–tandem mass-spectrometry and the Global Natural Products Social Molecular Networking. Ion was identified as [M+H]^+^ with -0.10 ppm mass error

**Figure S64**

Enterolactone identified in the rumen fluid of a non-lactating Holstein cow using ultra-high performance liquid chromatography–tandem mass-spectrometry and the Global Natural Products Social Molecular Networking. Ion was identified as [M+H]^+^ with 1.79 ppm mass error

**Figure S65**

12,13-DiHOME identified in the rumen fluid of a non-lactating Holstein cow using ultra-high performance liquid chromatography–tandem mass-spectrometry and the Global Natural Products Social Molecular Networking. Ion was identified as [M+H]^+^ with 0.43 ppm mass error

**Figure S66**

Glycerol 1-hexadecanoate identified in the rumen fluid of a non-lactating Holstein cow using ultra-high performance liquid chromatography–tandem mass-spectrometry and the Global Natural Products Social Molecular Networking. Ion was identified as [M+H]^+^ with 1.31 ppm mass error

**Figure S67**

13,14-Dihydro Prostaglandin F1a identified in the rumen fluid of a non-lactating Holstein cow using ultra-high performance liquid chromatography–tandem mass-spectrometry and the Global Natural Products Social Molecular Networking. Ion was identified as [M+H]^+^ with -1.53 ppm mass error

**Figure S68**

Niranthin identified in the rumen fluid of a non-lactating Holstein cow using ultra-high performance liquid chromatography–tandem mass-spectrometry and the Global Natural Products Social Molecular Networking. Ion was identified as [M+H]^+^ with -5.98 ppm mass error

**Figure S69**

7b,9-Dihydroxy-3-(hydroxymethyl)-1,1,6,8-tetramethyl-5-oxo-1,1a,1b,4,4a,5,7a,7b,8,9-decahydro-9aH-cyclopropa[3,4]benzo[1,2-e]azulen-9a-yl acetate identified in the rumen fluid of a non-lactating Holstein cow using ultra-high performance liquid chromatography–tandem mass-spectrometry and the Global Natural Products Social Molecular Networking. Ion was identified as [M+H]^+^ with -1.70 ppm mass error

**Figure S70**

Monensin B identified in the rumen fluid of a non-lactating Holstein cow using ultra-high performance liquid chromatography–tandem mass-spectrometry and the Global Natural Products Social Molecular Networking. Ion was identified as [M+Na]^+^ with 0.93 ppm mass error

**Figure S71**

Monensin identified in the rumen fluid of a non-lactating Holstein cow using ultra-high performance liquid chromatography–tandem mass-spectrometry and the Global Natural Products Social Molecular Networking. Ion was identified as [M+Na]^+^ with 2.42 ppm mass error

**Figure S72**

Monensin methyl ester identified in the rumen fluid of a non-lactating Holstein cow using ultra-high performance liquid chromatography–tandem mass-spectrometry and the Global Natural Products Social Molecular Networking. Ion was identified as [M+Na]^+^ with 1.74 ppm mass error
